# A Soft Tissue Osteochondrolipoma of the Lateral Ankle: A Case Report and Clinical Insight

**DOI:** 10.1155/cro/4209643

**Published:** 2026-07-27

**Authors:** Andi Praja Wira Yudha Luthfi, Bagus Iman Brilianto, Filbert Nathaniel, Weny Yusnita, Karina Sylvana Gani, Mitchel Mitchel, Erica Kholinne

**Affiliations:** ^1^ Department of Orthopedics and Traumatology, Bhayangkara Tk. I Pusdokkes Polri Hospital, Jakarta, Indonesia; ^2^ Department of Orthopaedic and Traumatology, Gatam Institute, Eka Hospital, Tangerang, Indonesia; ^3^ Department of Orthopaedic & Traumatology, Soeharso Orthopaedic Hospital, Surakarta, Indonesia; ^4^ Faculty of Medicine and Health Science Atma Jaya Catholic University of Indonesia, Jakarta, Indonesia; ^5^ Department of Pathological Anatomy, Bhayangkara Tk. I Pusdokkes Polri Hospital, Jakarta, Indonesia; ^6^ Department of Surgery, Faculty of Medicine, Universitas Trisakti, Jakarta, Indonesia

**Keywords:** ankle, cartilage, hyaline cartilage, lipoma, surgical excision

## Abstract

Tumors of the foot and ankle are uncommon, accounting for approximately 4%–10% of all musculoskeletal tumors; most are benign soft‐tissue lesions. Osteochondrolipoma is an exceptionally rare histological variant of lipoma characterized by mature adipose tissue with cartilaginous and osseous differentiation. It is typically reported in the head, neck, and upper extremities, whereas its occurrence in the lower extremity, particularly the hindfoot, is exceedingly rare. We report a case of osteochondrolipoma of the lateral aspect of the hindfoot of a 54‐year‐old female who presented with a painless, slowly enlarging mass causing difficulty with footwear. Imaging demonstrated a well‐defined, heterogeneous soft tissue lesion with ossified components, without bone attachment. Complete surgical excision was performed. Histopathological examination revealed mature adipose tissue interspersed with hyaline cartilage and mature bony trabeculae with evidence of endochondral ossification, confirming the diagnosis of osteochondrolipoma. The postoperative course was uneventful. At 3 months of follow‐up, the patient reported complete resolution of symptoms and no difficulty wearing footwear. Functional outcome improved, with the American Orthopaedic Foot and Ankle Society (AOFAS) Ankle–Hindfoot Score increasing from 81 preoperatively to 88. No clinical evidence of recurrence was observed. Osteochondrolipoma is a benign entity with an excellent prognosis after complete excision. Given its rarity in the hindfoot and its potential to mimic other calcified soft tissue tumors, clinicians and pathologists should consider osteochondrolipoma in the differential diagnosis of well‐circumscribed ossified masses of the foot and ankle.

## 1. Introduction

Bone and soft tissue masses are frequently seen in daily practice. However, musculoskeletal tumors of the foot and ankle are rare. Neoplasms in this anatomical region are predominantly benign [1]. Among mesenchymal tumors of the foot and ankle, lipomas are the most prevalent. Lipomas are composed of mature adipocytes and typically arise in the subcutaneous tissue [2]. Differentiation into other mesenchymal elements, including fibrous tissue, blood vessels, and muscle, has been documented. However, osseous or cartilaginous differentiation remains rare and is most often associated with a parosteal localization [3].

Previous studies have reported that osteochondrolipomas most commonly occur in the forearm, ischial region, mandible, axilla, scapular region, popliteal fossa, and chest wall [4, 5]. However, reports of osteochondrolipomas arising in the foot are limited in the literature. Consequently, the clinical characteristics, imaging features, and diagnostic considerations of osteochondrolipoma in the foot remain poorly defined, potentially leading to misdiagnosis when evaluating ossified soft‐tissue masses in this region. In this context, we present a rare case of osteochondrolipoma arising in the lateral hindfoot, detailing its clinical presentation, radiological findings, surgical management, and histopathological features. Our case has been reported in line with the CARE checklist [6].

## 2. Case Presentation

A 54‐year‐old woman presented with a 5‐year history of a gradually enlarging swelling over the lateral aspect of the right hindfoot. The mass was painless but progressively interfered with footwear use, particularly closed shoes. She denied the presence of similar lesions elsewhere in the body. The patient reported using various herbal medications in an attempt to reduce the swelling; however, no clinical improvement was noted. The patient had no significant past medical or surgical history and no known chronic illnesses or relevant comorbidities. She was not on any long‐term medication and reported no known drug allergies. There was no history of trauma, fever, fatigue, night sweats, unintentional weight loss, loss of appetite, or smoking. The physical examination revealed a firm, painless, nonfluctuant mass measuring approximately 5 × 4 cm that was palpated over the lateral aspect of the hindfoot (Figure 1). The overlying skin was intact, with no erythema, tenderness, or other superficial skin changes. The patient demonstrated full, pain‐free ankle range of motion, and neurological examination revealed no deficits. There was no regional lymphadenopathy. The preoperative American Orthopaedic Foot and Ankle Society (AOFAS) Ankle–Hindfoot Score was 81. The details of the timeline of this case can be seen in Table 1.

**Figure 1:**
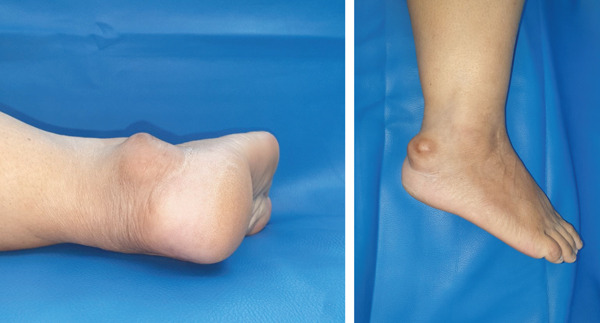
Clinical photograph demonstrating a firm, well‐defined swelling over the lateral aspect of the right hindfoot.

**Table 1:** The timeline of events.

Timeline	Clinical event
Five years before the presentation	Gradually enlarging painless swelling over the lateral aspect of the right hindfoot. The patient attempted herbal medications without improvement.
Preoperative	● Physical examination revealed a firm, nontender 5 × 4 cm mass with full ankle range of motion. Preoperative AOFAS Ankle–Hindfoot Score: 81.
	● Plain radiographs demonstrated a well‐circumscribed soft tissue mass with internal calcification.
	● MRI demonstrated a heterogeneous lesion with fatty, fibrous, and ossified components without continuity with the adjacent bone.
Operative	Complete excision of the well‐circumscribed soft tissue mass was performed. No attachment to the fibula, talus, or calcaneus was identified.
Histopathological examination	Histopathology confirmed osteochondrolipoma with mature adipose tissue, hyaline cartilage, mature bone, and endochondral ossification.
Two weeks postoperative	Uneventful wound healing with no signs of infection; sutures removed.
Four weeks postoperative	Returned to normal daily activities.
Three months postoperative	No clinical evidence of recurrence. AOFAS Ankle–Hindfoot Score improved from 81 to 88, with complete resolution of symptoms and no difficulty wearing footwear.

Abbreviation: AOFAS, American Orthopaedic Foot and Ankle Society.

Plain radiographs demonstrated a well‐defined soft tissue mass with areas of internal calcification (Figure 2). Magnetic resonance imaging (MRI) revealed a solitary, heterogeneous lesion measuring 2.32 × 2.96 × 2.95 cm, located lateral to the calcaneal wall, composed of mixed fatty, fibrous, and ossified components, without evidence of continuity with the adjacent bone (Figure 3).

**Figure 2:**
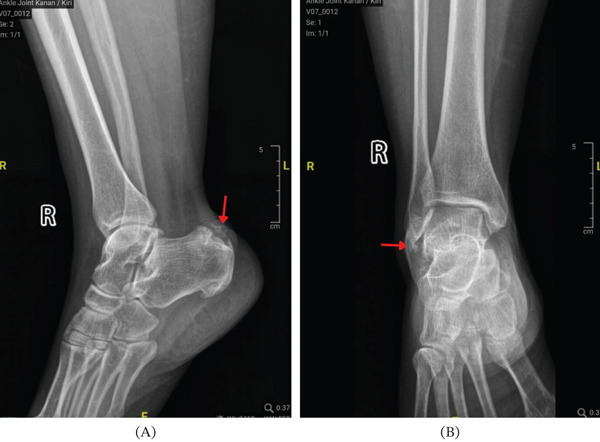
Plain radiographs of the right foot in (A) lateral and (B) anteroposterior views show a well‐circumscribed soft tissue mass with internal calcification (red arrow).

**Figure 3:**
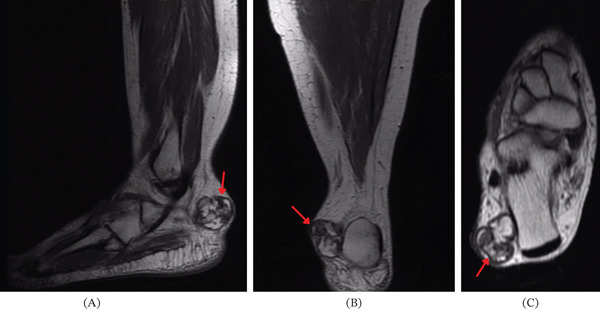
Magnetic resonance images of the right foot in (A) a sagittal, (B) a coronal, and (C) a transverse axial view show a lobulated, septated soft tissue mass with calcification.

Given the progressive symptoms and imaging findings, surgical excision was indicated. The procedure was performed under spinal anesthesia with the patient in the lateral decubitus position. A thigh tourniquet inflated to 350 mmHg was applied. Following skin incision and fascial dissection, a well‐circumscribed, solid mass was identified within the soft tissues. The lesion consisted of fibrous and chondral components and demonstrated no attachment to the fibula, talus, or calcaneus. Complete excision of the lesion (Figure 4) was carried out, followed by triple irrigation using hydrogen peroxide, iodine, and saline. The excised mass was submitted for histopathological examination to confirm the diagnosis. The wound was then closed in layers using standard surgical technique, and postoperative analgesics were administered. A solitary mass measuring 3.4 × 3.5 × 3 cm was excised (Figure 5A). Gross examination revealed mature fatty tissue with focal gray‐white areas corresponding to fibrous, cartilaginous, and osseous elements (Figure 5B).

**Figure 4:**
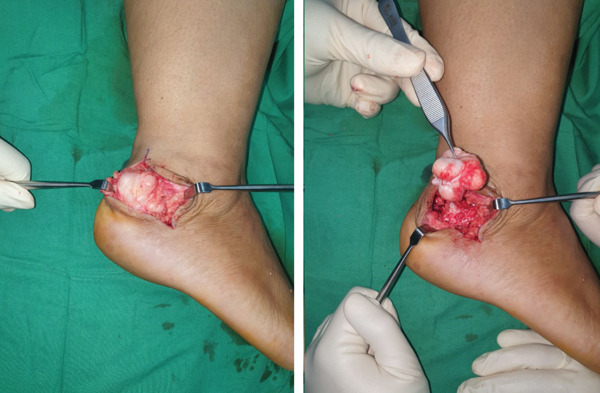
Intraoperative images demonstrating complete surgical excision of the well‐circumscribed hindfoot mass.

**Figure 5:**
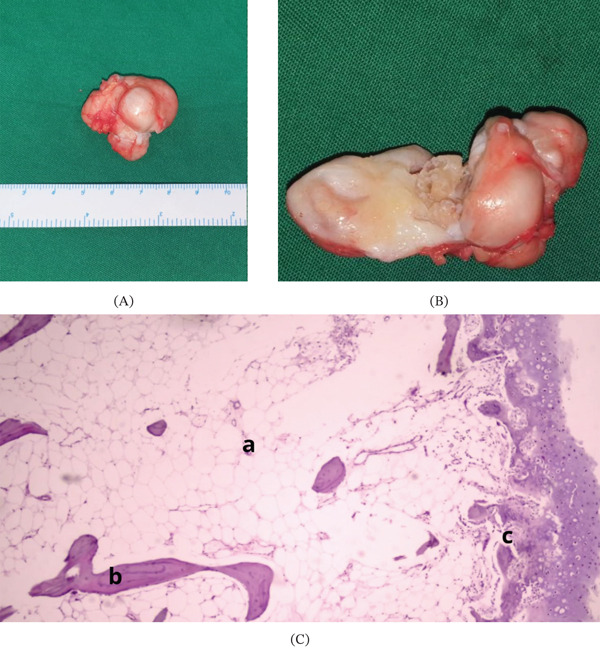
Gross and histopathologic findings. (A and B) Macroscopic examination of the surgical specimen and its sectioning revealed a solid, gray‐white, fleshy area. (C) Histopathological examination (hematoxylin and eosin staining, 100x) of the resected specimen revealed (a) lobules of mature adipose tissue of variable sizes interspersed with (b) bony trabeculae exhibiting osteoblastic rimming and (c) overlying mature hyaline cartilaginous tissue.

Histopathological examination revealed an encapsulated lesion composed of lobules of mature adipose tissue interspersed with bony trabeculae exhibiting osteoblastic rimming (Figure 5C). Foci of mature hyaline cartilage and areas of endochondral ossification were also identified. There was no evidence of hematopoietic tissue, cartilaginous cap formation, zonation, cellular atypia, mitotic activity, or fibromyxoid stroma, thereby excluding mesenchymal hamartoma, osteochondroma, myositis ossificans, well‐differentiated liposarcoma, and ossifying fibromyxoid tumor, respectively. These features were consistent with a diagnosis of osteochondrolipoma. Postoperatively, neurovascular status remained intact and no complications were observed. The patient was discharged home in stable condition with adequate pain control and a course of prophylactic antibiotics. At the 2‐week follow‐up, the surgical wound showed satisfactory healing, the sutures were removed, and there were no signs of infection or wound dehiscence. She resumed normal daily activities 4 weeks after surgery. At the 3‐month follow‐up, the patient reported complete resolution of symptoms, with no difficulty wearing footwear. Her AOFAS Ankle–Hindfoot Score improved to 88, and there was no clinical evidence of recurrence.

## 3. Discussion

Tumors of the foot and ankle are relatively rare. Previous studies have reported that lesions in this region account for approximately 5%–10% of all musculoskeletal tumors, as demonstrated by Toepfer et al. (5.5%) [1], Chou et al. (5.8%) [7], and Ozdemir et al. (10.9%) [8]. With regard to anatomical distribution, the hindfoot is the most frequently involved site for benign soft tissue tumors (28.7%), followed by the midfoot (25.9%), ankle (25.4%), and forefoot (20.0%) [9]. Within the hindfoot, the vast majority of lesions are soft tissue tumors (89.7%), whereas osseous tumors account for only 10.3% [10]. Importantly, malignant soft tissue tumors comprise merely 6.9% of hindfoot lesions. The most commonly encountered benign soft tissue tumors in this region include tenosynovial giant cell tumor, haemangioma, plantar fibromatosis, schwannoma, and lipoma [9].

Lipomas are among the most common benign soft tissue neoplasms and are characteristically slow‐growing lesions that may arise in nearly any anatomical location [11]. Histological differentiation into additional mesenchymal elements, such as fibrous tissue, vascular structures, or smooth muscle, has been well documented. However, differentiation into bone or cartilage is distinctly uncommon and is more frequently associated with parosteal localisation [12]. Osteochondrolipoma is therefore classified as a rare benign variant of lipoma, as osseous differentiation occurs in fewer than 1% of lipomas, and the coexistence of both cartilaginous and osseous components is even rarer [13].

Osteochondrolipoma is a benign lesion, as evidenced by the absence of cellular atypia, increased mitotic activity, and lipoblasts. In most reported cases, the lesion is well circumscribed, independent of the neurovascular bundle, and lacks direct attachment to the adjacent bone [14]. The majority of osteochondrolipomas described in the literature occur in the upper half of the body, including the submandibular region, tongue, intracranial interhemispheric and intratentorial regions, subcutaneous chest wall, and scapular region. Involvement of the lower extremity remains distinctly uncommon [13].

The pathogenesis of osteochondrolipoma remains unclear, and several theories have been proposed. One hypothesis suggests that adipose, cartilaginous, and osseous tissues arise from multipotent undifferentiated mesenchymal cells capable of divergent differentiation [15, 16]. Another theory proposes that cartilaginous and osseous components develop through a metaplastic process within a pre‐existing lipoma or its stromal tissue, potentially triggered by repetitive microtrauma or chronic mechanical stimulation leading to secondary ossification [12].

Clinical presentation of osteochondrolipoma varies considerably. Some cases describe lesions that are firm, mobile, and nonadherent to surrounding tissues, whereas others report masses that are firmly attached. Symptomatology ranges from painless swelling to pain or paresthesia, depending on tumor size, location, and proximity to neurovascular structures [12, 13, 15]. Given this variability, careful clinical assessment and establishment of a broad differential diagnosis are essential to avoid misdiagnosis.

Due to the compact anatomical structure of the foot, both osseous and soft tissue tumors are often detected early and are easily palpable. Even small lesions may cause pain or functional impairment, particularly during weight‐bearing or footwear use [7]. However, clinical features such as pain, lesion size, or history of trauma do not reliably correlate with malignancy [8]. Although early detection may theoretically aid in identifying malignant lesions, the low prevalence of malignancy in this region frequently leads to diagnostic uncertainty and, in some cases, inappropriate management [8, 9].

Imaging plays a critical role in the evaluation of osteochondrolipoma. Plain radiographs typically demonstrate mineralized or ossified components, while computed tomography (CT) is valuable for delineating the coexistence of fatty and osseous elements and for assessing the relationship between the lesion and adjacent bone [12]. In the present case, CT clearly demonstrated peripheral ossification and confirmed the absence of bony continuity. Magnetic resonance imaging (MRI), however, remains the imaging modality of choice for adipocytic tumors. Simple lipomas typically show homogeneous signal intensity identical to subcutaneous fat on all sequences, with complete signal suppression on fat‐suppressed images. In contrast, areas of ossification, calcification, and fibrous tissue appear as regions of low signal intensity across all MRI sequences [17–19].

Definitive diagnosis of osteochondrolipoma relies on a combination of imaging findings and histopathological examination. Comprehensive histological evaluation of the entire lesion is crucial to minimize the risk of misdiagnosis. Histologically, osteochondrolipoma appears as a well‐encapsulated lesion containing mature adipose tissue interspersed with cartilaginous and osseous components, surrounded by a vascularized fibrous capsule [15]. Microscopic examination often reveals osteocytes with osteoblastic rimming consistent with woven bone formation, with cartilaginous tissue typically located adjacent to ossified areas [13].

Despite the characteristic admixture of adipose, cartilaginous, and osseous tissues, alternative diagnoses must be carefully excluded. Cartilaginous differentiation may also be seen in extraskeletal chondroma, chondroid lipoma, or posttraumatic chondrification [16, 17]. The differential diagnosis of calcified soft‐tissue masses includes myositis ossificans, ossifying fibromyxoid tumor, osteoma, secondary hyperostosis, dedifferentiated liposarcoma, osteosarcoma, chondrosarcoma, and teratoma. Ultimately, accurate diagnosis depends on careful assessment of cellular atypia, pleomorphism, and the architectural relationships among tissue components, including their proportions, encapsulation, and anatomical locations [13.14].

Complete surgical excision remains the treatment of choice for osteochondrolipoma. Needle biopsy is often insufficient due to limited sampling and inability to evaluate the overall tumor architecture or assess local invasion. Consequently, incisional biopsy or complete excision is preferred to allow thorough pathological examination. Submission of the entire specimen for histopathological analysis is strongly recommended to confirm the diagnosis and exclude malignant entities [2, 5]; osteochondrolipoma carries an excellent prognosis comparable to that of a simple lipoma. To date, neither local recurrence nor malignant transformation has been reported following complete excision [4, 20].

The primary strength of this case report is the presentation of an extremely rare osteochondrolipoma located in the hindfoot, supported by detailed clinical evaluation, imaging findings, surgical management, histopathological confirmation, and functional outcome assessment using the AOFAS Ankle–Hindfoot Score. Nevertheless, the limitations include its single‐case nature and a relatively short follow‐up duration, which restrict evaluation of long‐term functional outcomes and recurrence.

## 4. Conclusion

Osteochondrolipoma is an exceptionally rare benign variant of lipoma that may occur in the hindfoot and present as a slowly enlarging, painless soft tissue mass causing functional impairment. This case highlights the diagnostic challenge posed by ossified soft tissue lesions in the foot and ankle, where clinical features alone are insufficient to distinguish benign from malignant pathology. Careful imaging evaluation combined with complete surgical excision and thorough histopathological examination is essential for accurate diagnosis. Awareness of osteochondrolipoma as a potential differential diagnosis for well‐circumscribed, calcified or ossified hindfoot masses can help prevent misdiagnosis and ensure appropriate management. Complete excision provides both definitive diagnosis and excellent clinical outcomes.

## Funding

No funding was received for this manuscript.

## Ethics Statement

Ethical approval for the case report was granted an exemption by the ethical committee of the Faculty of Medicine, University of Trisakti, on January 10, 2026.

## Consent

Written informed consent for publication was obtained from the patient and/or legal guardian.

## Conflicts of Interest

The authors declare no conflicts of interest.

## Supporting information


**Supporting Information** Additional supporting information can be found online in the Supporting Information section. Our case has been reported in line with the CARE checklist.

## Data Availability

The data that support the findings of this study are available on request from the corresponding author. The data are not publicly available due to privacy or ethical restrictions.
